# 449. Further Evidence for Subphenotype E of Staphylococcus aureus Bacteremia (SAB) in People who Inject Drugs (PWID)

**DOI:** 10.1093/ofid/ofaf695.148

**Published:** 2026-01-11

**Authors:** Elena Milin, Yoseph Aldras, Stephanie Spivack

**Affiliations:** Lewis Katz School of Medicine at Temple University, Philadelphia, Pennsylvania; Temple University Hospital, Philadelphia, Pennsylvania; Temple University Health System, Philadelphia, PA

## Abstract

**Background:**

*Staphylococcus aureus* bacteremia (SAB) is associated with a mortality rate of 25-30% within 3 months. In 2024, Swets et al. described 5 distinct clinical subphenotypes of SAB. Subphenotype E was associated with community-acquired SAB in PWID and had the highest 1-year survival rate.

**Figure 1:**
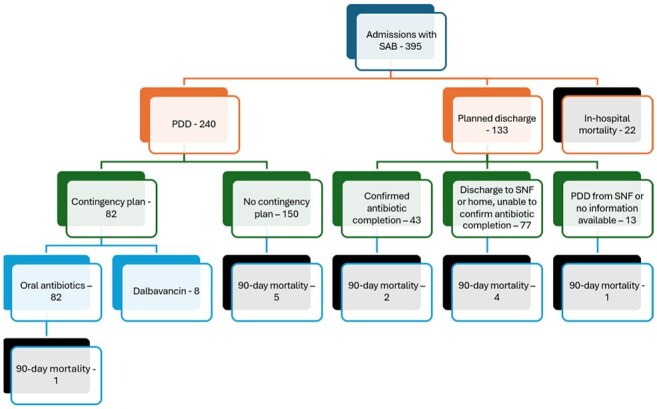
Treatment and outcomes of patients with SAB.

**Figure 2:**
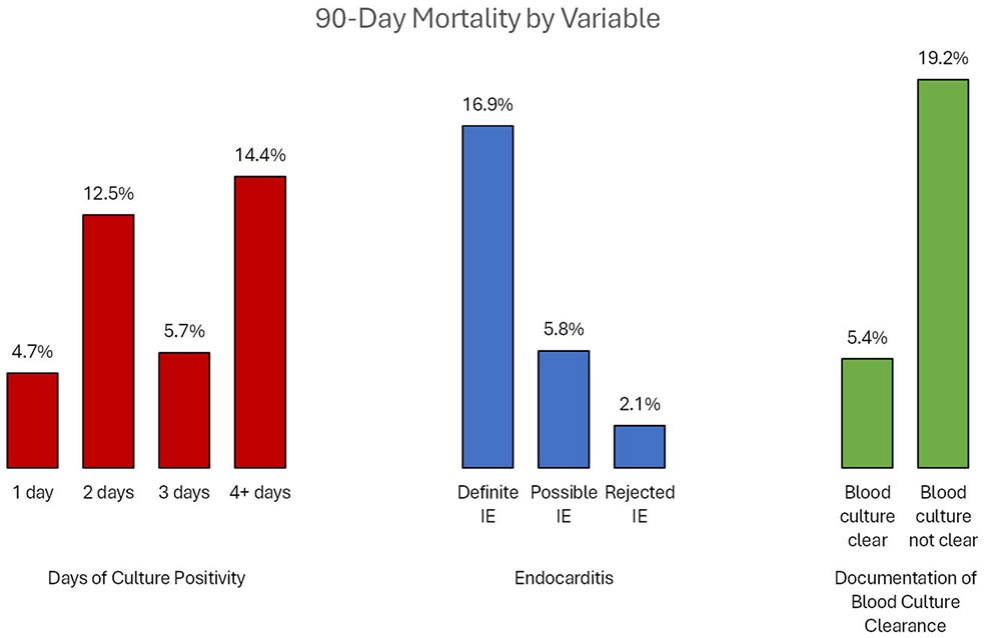
Mortality stratified by days of blood culture positivity, classification of endocarditis by modified Duke criteria, and documentation of blood culture clearance before discharge.

**Methods:**

We performed an IRB-approved retrospective chart review of hospitalized PWID with positive blood cultures between March 2022 and March 2024.

**Results:**

A total of 382 patients had 574 admissions with positive blood cultures; of these, 280 patients had 395 admissions with SAB. The median age was 38, 45% were female, and 77% had unstable housing. 81% had methicillin-susceptible *Staphylococcus aureus* (MRSA) and 22% had methicillin-susceptible *Staphylococcus aureus* (MSSA); 13 patients had both.

Overall, 22 patients with SAB had in-hospital death and an additional 13 died within 90 days (22% mortality).

More than half (240, 61%) of admissions ended with a patient-directed discharge (PDD), while 133 (34%) discharges were planned. A contingency plan was used in 90 PDDs (23%); 82 were given oral antibiotics and 8 received dalbavancin. We could confirm that the first-line antibiotic course was completed in only 43 cases (11%); 2 died within 90 days (5% mortality). (Figure 1)

By modified Duke criteria, 124 of 395 admissions (31%) had definite infective endocarditis (IE), 224 (57%) had possible IE, and 48 (12%) had rejected IE. In 272 cases with possible or rejected IE, 6 had in-hospital mortality and 7 died within 90 days (5% mortality).

Blood cultures were positive for only 1 day in 191 (48%) of admissions; in this group, 5 had in-hospital death and 4 died within 90 days (5% mortality). There was documented clearance of blood cultures before discharge in 294 admissions; in this group, 7 had in-hospital mortality and 9 within 90 days (5% mortality) (Figure 2).

**Conclusion:**

In this cohort of PWID with SAB, the mortality rate is lower than that reported in the literature. This rate is low despite high rates of PDD and low rates of antibiotic completion. Mortality is especially low in patients who did not have definite IE and those who had only 1 day of blood culture positivity, had culture clearance before discharge, or completed treatment.

**Disclosures:**

All Authors: No reported disclosures

